# Mental Health Providers’ Challenges and Solutions in Prescribing Over Telemedicine: Content Analysis of Semistructured Interviews

**DOI:** 10.2196/65419

**Published:** 2025-03-20

**Authors:** Julia Ivanova, Mollie R Cummins, Hiral Soni, Triton Ong, Brian E Bunnell, Esteban López, Brandon M Welch

**Affiliations:** 1 Doxy.me Research Doxy.me Inc Charleston, SC United States; 2 Department of Biomedical Informatics, College of Nursing and Spencer Fox Eccles School of Medicine, University of Utah Salt Lake City, UT United States; 3 Department of Psychiatry and Behavioral Neurosciences, Morsani College of Medicine, University of South Florida Tampa, FL United States; 4 Biomedical Informatics Center, Medical University of South Carolina Charleston, SC United States

**Keywords:** telemedicine, telehealth, prescribe, prescription, drug, pharmacology, pharmacotherapy, pharmaceutical, medication, barrier, buprenorphine, mental health, digital health, informatics, qualitative analysis, content analysis, provider perspective, provider, experience, attitude, opinion, perception, perspective

## Abstract

**Background:**

In response to the COVID-19 pandemic, the United States extended regulatory flexibilities to make telemedicine more accessible to providers and patients. Some of these flexibilities allowed providers to intake patients over telemedicine and prescribe certain scheduled medications without an in-person visit.

**Objective:**

We aim to understand providers’ parameters for their comfort in prescribing over telemedicine and report on solutions providers have adopted in response to potential barriers and challenges in prescribing via telemedicine.

**Methods:**

As part of a larger mixed methods study between February and April 2024, we conducted 16 semistructured interviews with mental health providers who prescribe via telemedicine within the United States. We used the results of a web-based, cross-sectional survey to develop a codebook and support recruitment. We analyzed a subsection of the 16 interviews using content analysis to capture comfort, barriers, and workarounds in telemedicine prescribing. We reported codes by frequency and by provider.

**Results:**

Participants were typically male (11/16, 69%), provided care mostly or completely over telemedicine (11/16, 69%), and were psychiatrists (8/16, 50%) or other physician (3/16, 19%). Providers’ primary states (10/16, 62%) of practice included Oregon, Texas, New York, and California. The content analysis yielded a total of 234 codes, with three main codes—comfort (98/234, 41.9%), barriers or challenges (85/234, 36.3%), and workarounds or solutions (27/234, 11.5%)—and two subcodes—uncomfortable prescribing (30/98, 31%) and comfortable prescribing (68/98, 69%) over telemedicine. Participants reported being comfortable prescribing over telemedicine as long as they could meet their main parameters of working within their expertise, having access to needed patient health information, and being compliant with rules and regulations. Participants reported frustrations with e-prescription workflows and miscommunications with pharmacies. Solutions to ease frustrations and alleviate discomforts in prescribing over telemedicine included developing workflows to help patients complete laboratory tests and physical examinations and directly communicating with pharmacies.

**Conclusions:**

By applying content analysis to the semistructured provider interviews, we found that physicians are comfortable prescribing via telemedicine when they feel they are practicing within their personal parameters for safety. While many providers experience frustrations such as miscommunication with pharmacies, these barriers appear to not prevent them from telemedicine prescribing. With expected changes in 2024 and 2025 to the US laws and regulations for telemedicine prescribing, we may see changes in provider comfort in prescribing.

## Introduction

During the height of the COVID-19 pandemic, health care visits moved to telemedicine when possible to help stem the spread of infection [1]. In the United States, the public health emergency (PHE) and regulatory flexibilities were instrumental in streamlining this effort by waiving specific requirements, such as the providers’ ability to see patients outside the states where they are licensed and to prescribe over telemedicine with fewer restrictions [2,3]. Due to these temporary changes, providers could prescribe via telemedicine less restrictively and determine what level of care they felt comfortable providing over telemedicine.

The US Drug Enforcement Administration extended prescribing flexibilities past the May 11, 2023, PHE deadline and is in the process of finalizing a rule regarding telemedicine prescribing [4,5]. There has been concern that waiving elements of the Ryan Haight Act, which previously required providers to meet with patients in person before prescribing certain controlled substances, would adversely impact patient outcomes [6,7]. The concurrent opioid epidemic has also provided a new level of scrutiny in the space of telemedicine and prescribing scheduled medication for the treatment of substance use disorder (SUD) [8-10]. As US providers await the promulgation of this final rule, evidence continues to grow regarding positive patient outcomes via mental health prescribing over telemedicine [11]. One Medicaid data study following over 90,000 patients showed remarkable positive outcomes from telemedicine initiation of buprenorphine treatment, including better odds of 90-day treatment retention than if initiation occurred in person [12]. In a scoping review from 2008 to March 18, 2021, reviewers found that telehealth technology in SUD treatment increased access and adherence to buprenorphine and generally showed higher patient satisfaction and comparability to in-person retention rates [11]. Especially within the space of buprenorphine prescription, evidence supports that telemedicine offers comparable treatment to in-person care and can be an effective method for increasing patient access to mental health treatments [8,11].

With the flexibilities, providers can now choose the extent to which they use telemedicine. One study reported psychiatrists felt telemedicine use in a hybrid scenario allowed for treatment adaptation based on client needs and access [13]. The study’s participants emphasized their need to consider each patient’s unique case in determining whether they could successfully leverage telemedicine to initiate care or ensure continued visits over time [13]. For example, one psychiatrist in the study noted the following:

When you have these patients who are at risk for falling out of care, if you don’t offer them telemedicine, but [they] are also at risk for getting slightly suboptimal care when you do offer them telemedicine, it is a very case-by-case judgment call in terms of the risks and benefits of enabling the telemedicine.

Such sentiments support the call for greater provider autonomy and options, as well as the focus on including telemedicine as part of the holistic health care model [13-15].

Though there is evidence supporting the safety of prescribing certain scheduled substances over telemedicine (even without an initial in-person visit) [8,11,12], there is a need for greater insight into mental health providers’ experiences in prescribing scheduled medications during and after the PHE. A better understanding of providers’ perspectives, experiences, and comfort in prescribing for their patients over telemedicine would build a more accurate understanding of what providers view as actual challenges and limitations. This knowledge would add to the discourse regarding updates in policy and guidelines, especially necessary for policy makers as they deliberate over proposed rules and regulations [16]. To the best of our knowledge, no previous studies have focused on mental health providers’ experiences regarding general prescribing—including scheduled medications—over telemedicine. In early 2024, we conducted a mixed methods study that included a web-based, cross-sectional survey and semistructured interviews of mental health providers who prescribe via telemedicine. The survey aimed to assess provider perceptions, experiences, comfort, and perceived patient safety in prescribing medications over telemedicine [17]. The interviews aimed to get a better understanding of providers’ general telemedicine use, challenges and solutions specific to prescribing over telemedicine, and providers’ knowledge and experiences regarding compliance with prescribing over telemedicine. In this study, our objectives were (1) to understand better providers’ parameters for safe prescribing over telemedicine and (2) to report practices or workflows providers have adopted to accomplish safe prescribing via telemedicine. The results of the study will help stakeholders reassess long-standing policies—made at the beginning of these technological advances—that may now be causing unintended harm as the technology has improved and become more accessible to laypeople.

## Methods

### Study Design

We performed exploratory content analysis of semistructured interviews with tele–mental health care providers as part of a larger, mixed methods study. We used an explanatory sequential design, building upon a previously reported, web-based, cross-sectional survey [17].

### Interview Development

After an initial literature review, we developed the semistructured interview guide with consultation and pretesting from 5 providers. We estimated that interviews would take 60 minutes. The interview attempts to capture context regarding what factors affect providers’ views and comfort in prescribing medications via different scenarios and combinations of in-person and telemedicine visits. The interview guide covers the topics of (1) the provider’s practice, such as specialty, telemedicine use, patients, and general difficulties with telemedicine; (2) challenges and solutions concerning prescribing over telemedicine; and (3) knowledge pertaining to laws and regulations of prescribing to telemedicine (see Multimedia Appendix 1 for the interview guide). This study focuses specifically on answers to the challenges and solutions concerning prescribing over telemedicine (Multimedia Appendix 1).

### Participants and Recruitment

We recruited participants for the semistructured interviews from among participants in a research survey. The survey sampling, recruitment, and results are reported elsewhere [17]. In brief, we recruited a sample of US tele–mental health care providers from a telemedicine research panel, TelehealthEngage [18]. A total of 115 participants fully completed the survey. Participants were predominantly White (83/115, 72.2%), non-Hispanic or Latino (97/115, 84.3%), female (66/115, 57.4%) providers who were physicians (69/115, 60%) seeing their clients most to all of the time (50%-100% of visits) over telemedicine (82/115, 71.3%) [17].

Participants who agreed to be contacted at the end of the survey were invited to participate in follow-up interviews. Of the 115 completed surveys, 59 (51.3%) participants showed interest in being interviewed. Research team member JI (a White, immigrant female individual with a background in medical anthropology, psychology, and biomedical informatics) sent out interview invitations and completed interviews between February and April 2024. We aimed to complete 20 interviews or until we reached content saturation [19].

### Data Analysis

One author (JI) performed an exploratory content analysis of 6 interviews. The codebook for content analysis, consisting of three main codes and two subcodes, was specified on the basis of prior survey findings (Table 1) [17]. The codebook was developed to test our expectations that providers will feel comfortable when their personal parameters for patient safety have been met and when the telemedicine visit is within the scope of provider practice and expertise. We used content analysis of the interviews to identify codes related to personal parameters for comfort in prescribing over telemedicine, named barriers and challenges of telemedicine prescribing, and the types of workarounds providers have found useful within those situations [19].

**Table 1:** Demographics as reported by providers during the interview^a^.

Demographics for content analysis		Value, n (%)
**Sex (n=16)**		
	Male	11 (69)
	Female	5 (31)
**How often telemedicine is used (n=16)**		
	None to seldom	1 (6)
	Fairly often (about half the time)	4 (25)
	Mostly or completely	11 (69)
**Provider type (n=16)**		
	Psychiatry	8 (50)
	Family medicine or general practice physician	3 (19)
	Nurse practitioner	3 (19)
	Physician assistant	2 (13)
**Type of practice (n=21)**		
	Private practice	12 (57)
	Statewide health system	4 (19)
	Community health center	2 (10)
	Solo practice	1 (5)
	City-run program	1 (5)
	Outpatient rehabilitation center	1 (5)

^a^Some demographic values sum to more than 16 as some providers worked in multiple practices.

We transcribed and deidentified completed interviews using Dovetail software (Dovetail). We confirmed transcripts for accuracy prior to deleting video data of interviews and uploaded transcripts to MAXQDA 2024 software (MAXQDA) for analysis. A single author (JI) coded the interviews over three iterations using the previously developed codebook, and coauthor HS (an Asian, immigrant female individual with a background in biomedical informatics and human factors research in behavioral health) reviewed the codes and codebook for validity and accuracy. We resolved discrepancies through consensus to ensure consistency and accuracy in the qualitative method [20,21]. The unit of analysis was meaningful phrases. Although we calculated the total frequency of code instances, we primarily focused on the frequency of codes by individual participants [19]. Additionally, contextual subtopics within the main reported codes were often double coded—as participants provided multiple examples, explanations, and reasoning—resulting in percentages over 100. We reported participant characteristics in the aggregate to ensure participants’ confidentiality. For context, we included participants’ numbers (1 through 16) after each quote.

### Ethical Considerations

This study was reviewed and approved by the BRANY Institutional Review Board (IRB00010793). We attained verbal informed consent for the interviews in addition to the written informed consent from the survey protocol. Participants had the opportunity to opt out of our research. Participants were compensated with a US $75 e-gift card for their time. While there is always a risk of selection and other compensation-related biases, the amount was deemed appropriate for US mental health professionals who were the focus of recruitment. We use deidentified data for the purposes of this research.

## Results

### Demographics

We completed 16 semistructured interviews between February 22 and April 26, 2024. There were 11 (69%) male and 5 (31%) female participants (see Table 1 for demographics). Participants primarily practiced in the following states: Oregon (n=3, 19%), Texas (n=3, 19%), New York (n=2, 12%), Illinois (n=2, 12%), California (n=1, 6%), Florida (n=1, 6%), Michigan (n=1, 6%), Washington (n=1, 6%), Indiana (n=1, 6%), and Tennessee (n=1, 6%). The majority (n=11, 69%) provided care mostly or completely over telemedicine (75%-100% of visits), 4 (25%) providers reported an even mix (40%-60% of telemedicine and in-person visits) in their hybrid practice often depending on the weather, and 1 (6%) provider reported almost complete in-person care except for emergencies. Providers included psychiatrists (n=8, 50%), family medicine or general practice physicians (n=3, 19%), nurse practitioners (n=3, 19%), or physician assistants (n=2, 12%). Providers reported working in private practice (n=12, 75%), statewide health systems or clinics (n=4, 25%), community health centers (n=2, 12%), a solo practice (n=1, 6%), a city-run program (n=1, 6%), and an outpatient rehabilitation center (n=1, 6%). Some providers worked in multiple positions or practice types.

### Content Analysis

A total of 234 code instances were identified, corresponding to three main codes and two subcodes. Table 2 shows the frequency of code instances corresponding to each main code and subcode. Frequencies of instances of *generally uncomfortable* and *generally comfortable* subcodes add up to the total frequencies of the *comfort* main code instances.

Of the 98 (41.9%) out of 234 total code instances under *comfort* in prescribing medications over telemedicine, 69% (68/98 code instances) were related to describing positive comfort in prescribing from all 16 providers. However, the majority of providers (10/16, 62%) also reported varying discomfort (30/98, 31% code instances) in prescribing over telemedicine even though it was less frequently mentioned.

Providers reported (85/234, 36.3%) varied barriers and challenges (85 code instances, 14 providers), providing information regarding problems that both providers and their patients face when prescribing medications over telemedicine. Providers reported fewer (27/234, 11.5%) workarounds and solutions (27 code instances, 11 providers).

**Table 2:** Content analysis results with the frequency of instances by main codes and subcodes^a^.

Name	Frequency of code instances, n	Definition	Example
Comfort (main code)	98	Any direct discussion regarding feelings of comfort or ease in prescribing over telemedicine	“I just don’t wanna like, yeah, miss something. And like, actually probably more so with the Benzos than with, than with stimulants because I feel like the stimulants you can: see if people are kind of like … when people are on Benzos they might be more drowsy, that you’re just not picking up with, on the, the, the, the virtual…” [Participant 1]
Generally uncomfortable (subcode)	30	Any direct discussion regarding feelings of discomfort or unease in prescribing over telemedicine	“I feel less comfortable with over telemedicine because sometimes just like the cadence of a conversation is hard. Sometimes if there’s a lag in telehealth or like whatever it may be. As for prescriptions, I would say, like your level twos and level threes are ones that I would not consider prescribing unless I’d at least seen them once in person…” [Participant 3]
Generally comfortable (subcode)	68	Any direct discussion regarding positive feelings of comfort or ease in prescribing over telemedicine	“And I would say that it’s really about the same, the actual prescribing is pretty darn smooth. So, I feel like the mechanics of the prescribing are the same. I do not prescribe differently because I, I’ve got a sense, I don’t know this person as well because I’m not me. No, I feel like I know my patients really well. Certainly, before I’m gonna be prescribing, I know what the story is in my mind. I may be wrong, but I’ve got that level of certainty. So, I don’t think the telehealth platform impacts my decision-making or the mechanics around prescribing significantly.” [Participant 6]
Barriers and challenges (main code)	85	Any direct discussion where a provider mentions a problematic situation that affects their decision-making to prescribe over telemedicine or affects a patient’s ability to receive treatment	“But I would say, yes, it’s difficult to feel comfortable, directing someone in another state to something that they may need without really knowing where that resource, who that resource is.” [Participant 7]
Workarounds and solutions (main code)	27	Any direct discussion where the provider describes a way they adapt their process or decision-making due to a barrier or challenge	“So, a lot of times I give them information on how to find a particular how to vet another practitioner, but that’s about the best [I can do].” [Participant 7]

^a^Main code and subcode definitions were developed based on survey analysis.

### Parameters for Comfort in Prescribing Over Telemedicine

#### Overview

Providers brought up three main parameters as they contextualized their comfort or discomfort with prescribing medications over telemedicine: (1) limits of telemedicine and personal expertise, (2) knowledge of patients and access to their relevant health information, and (3) liability concerns.

#### Limits of Telemedicine and Personal Expertise

Five (31%) of the 16 providers established that they felt comfortable when the interaction stayed within their perceived limits of telemedicine and occurred within their realm of expertise. One provider pointed out that these parameters essentially establish criteria for a successful telemedicine visit:

So the direct answer to the question is I feel very comfortable [prescribing over telemedicine]. But I think the reason that I feel very comfortable has to do with the patients: who I see and who I won’t see on telemedicine. So again, doing this is like a philosophical thing. I’m a big believer in getting people to the right place. And a lot of times that’s me and a lot of times that’s not me when it comes to mental health and telehealth, I try to, there are things that I, I don’t personally feel comfortable or think would be appropriate to, to treat. So in general, if someone is like in a manic state, if they’re psychotic, if they’re actively suicidal, like suicidal with a plan intending to act on it, that sort of stuff, I won’t see them through telehealth or if I have a patient who starts to experience those things, I will refer them for a higher level of care and we’ll be pretty insistent on it. Not that I’m not gonna see them anymore, but it’s just I want to get you to the best place.

Participant 13

Regarding the parameter of experience and expertise, 3 (19%) providers reported feeling uncomfortable prescribing over telemedicine when they thought they did not have the expertise to treat a particular diagnosis. One provider described feeling nervous about such a scenario:

I don’t have anybody that I’m treating for narcotic abuse or opiate stuff. So I, not that I wouldn’t, but I don’t have anybody and I don’t have a lot of experience with that. So that would make me nervous, especially over telemed. I just because I don’t do it much in person either.

Participant 9

In this case, the provider notes they would feel uncomfortable treating such a patient in person and that seeing such a case over telemedicine would increase that feeling.

#### Knowledge of Patients and Access to Their Relevant Health Information

This parameter encompassed several topics including working with established patients and accessing needed patient health information such as labs or physical exams. A total of 4 (25%) providers identified knowledge of a patient’s history—especially in regard to them being an established patient—as a crucial element of feeling comfortable prescribing for them.

So, certain medication that requires good monitoring for that level that may make me not want to prescribe medications on this platform. But I don’t think it has anything to do with virtual platforms, any providers or psychiatrists would do so in, in person also. So I’m not, I’m trying to think if, unless the only time I don’t prescribe is if someone makes the first appointment and say I’m on this medicine and I need refill, I don’t do that because that’s not a good practice. It’s like, OK, unless I have some data or previous records or not a very strong medication, then you can continue. But if someone comes with a specific request for medicines that are not appropriate, then I wouldn’t consider that as an OK to do it as a virtual practice.

Participant 10

This provider noted that in the scenario provided, they would need more information about the patient before prescribing, regardless of whether the visit is in person or via telemedicine. Meanwhile, one of these providers mentioned the importance of generally knowing their patients and their health status as they determine whether telemedicine visits are sufficient for care:

...If you have somebody who’s a fragile diabetic, you need to have a different level of observation for that person. But if you have a stable diabetic, then you can just continue to prescribe that whatever it might be…

Participant 15

Inherent in this explanation is that the provider felt they have sufficient knowledge regarding a patient’s current and past health status and feeling personally able to diagnose and treat in the presenting situation.

A total of 4 (25%) providers commented on the importance of having established patients in a scenario to make providers feel more comfortable prescribing over telemedicine. One of these providers underlined the importance of having such a relationship, especially when seeing patients for SUD treatment, as they may be able to pick up on potential concerns requiring more oversight via telemedicine or in person:

I mean, people with substance use problems. Now that brings a whole host of challenges itself because of confidence in, in history, you know, and confidence of just general reporting ... So I have a special, I have a different kind of relationship with [specific patient with SUD]. I can tell when somebody’s bullshitting. And I can call them pretty well on it. I rarely get blindsided because I’ve been doing this for a long time. So I come with enough experience, I think also that allows that to happen. But suffice to say that if I have an issue with someone there, I’ll put more controls over it, and we’ll go to task if needed. If they need to come in, I need to see them in person...

Participant 15

In this case, the provider leveraged not only their years of experience in the specific field (expertise parameter) and having known the patient for a long time but also their hybrid practice, as they can have the patient come for an in-person visit.

A total of 3 (19%) providers mentioned they feel comfortable prescribing over telemedicine because they have such an option. In addition to this, 4 (25%) providers also pointed out that having a way to check on their patient’s safety, whether it be having local resources for the patient or being able to view a Prescription Drug Monitoring Program, influences their comfort positively. One of these 4 providers described that knowing they can get patient health information quickly and seamlessly made them comfortable prescribing for an individual:

If they have established care, either someone who can update me on their vitals or regular monthly visits and have some kind of formal evaluation for me to backtrack the diagnosis. And then there is a way to order urine drug screens to make sure they’re not abusing any other drugs on the top of. So, if that makes it an easy flow for us, then, yeah. Yeah. I think that’s not a big problem. It’s just how to coordinate that care sometimes makes it harder...

Participant 10

Additionally, 4 (25%) providers stated the importance of developing and adhering to a protocol with their patients. Providers discussed this concept within the patient information parameter in everything from the formal quality of care processes to simply expecting to see a patient in person for all intakes. Adhering to protocols appears to be driven by the need for patient health status and history, as providers often discussed continuity of care and procurement of labs and exams.

So, I think patients who are, really complex, I feel less comfortable with prescribing over telemedicine because sometimes just, like, the cadence of a conversation is hard. Sometimes if there’s a lag in telehealth or whatever it may be. As for prescriptions, I would say, your level two’s and level three’s are ones that I would not consider prescribing unless I’d at least seen them once in person. I know personally, our clinic does random drug tests on our patients...

Participant 3

#### Liability Concerns

A total of 5 (31%) providers noted that they maintain their comfort in prescribing over telemedicine by simply refusing to prescribe certain controlled substances or not accepting specific diagnoses in their practice, especially due to their wariness of laws and regulations. One of these providers explained that while they treat SUDs, they do not prescribe certain medications and are aware of prescribing barriers:

...I do see a lot of patients with substance use disorder...just the fact that it’s [buprenorphine] more accessible now is amazing for these patients. I’ve heard there are still some barriers with methadone in particular...it hasn’t been an issue for me because I don’t prescribe that medication in particular.

Participant 16

Indeed, 2 (12%) providers indicated they avoid prescribing over telemedicine unless they feel they have to for the patient.

I generally refrain from prescribing anything scheduled over telemedicine ... just because of the, the insane liability with it ... the unbalanced liability, I would say that physicians shoulder in this industry.

Participant 7

Here, the provider also touched on the issue of compliance and liability inherent in this situation.

### Barriers and Workarounds in Prescribing Over Telemedicine

Of the 85 (36.3%) out of the 234 total code instances that describe barriers (14 providers), 37 (44%) of the 85 instances specifically mentioned that the e-prescription platform and the pharmacies caused challenges in prescribing medicines or picking up medicines (13 providers). There were 17 (20%) instances of providers describing difficulties with the actual e-prescription platform they use.

But Epic, they started putting in all these like hard blocks like you need to click this before you can do what you’re trying to...I think the way that they went about it sometimes, it was really like a hassle for the workflow when you add it all together.

Participant 2

Providers also often noted their patients’ prescription order was not received or not fulfilled by the pharmacy due to shortages in medicines or misunderstandings in prescribing over telemedicine (20/85, 24%). One provider noted how such a situation acted as a barrier for patients receiving their necessary prescriptions:

But I had a pharmacy once, call me that said that...they weren’t going to allow my patient to fill a pack of the prescription because I hadn’t provided an estimated glomerular filtration rate or creatinine clearance with my prescription. But like that’s because they now have the ability to prescribe that on their own. But I sent the prescription. I’m assuming the responsibility. I already checked that all you’re doing is throwing up another barrier to this patient, getting their medication when you do that, which I get. You don’t want to be audited or whatever, but you’re not the one prescribing it. I am, you know what I mean? I just like the pendulum has swung so far the other way since the opioid epidemic that they’re really trying to tighten the noose and on all this stuff, and I just think it’s going to end up harming patients who are calling for their beta blockers or, you know, whatever.

Participant 2

Providers reported shortages of certain medicines, usually stimulants, in pharmacies, requiring patients to find pharmacies with stock and providers to resend prescriptions. Providers’ complaints regarding the e-prescription platforms and pharmacies’ inability to provide the medications ranged from workflow nuisances to concerns that their patients could not receive necessary medications promptly.

Other mentioned barriers (49/85, 58%) were related to general telemedicine concerns and issues such as patient health concerns (17/85, 20% codes; 11/16, 69% providers), rules and regulations (16/85, 19% codes; 7/16, 44% providers), and reimbursement concerns (9/85, 11% codes; 2/16, 13% providers). Patient safety concerns mainly touched on worries that patients are not having their follow-up appointments, exams, and labs in a timely manner, which can affect patient health outcomes, as well as the ability of providers to prescribe medications for them.

I will put a child on a medication and I’ll say I need to have you call me in two or three days because this medicine doesn’t take a long time to start working. And I need to know so we can make adjustments. And invariably 99% of the time they wait till the next month, scheduled appointment and we could have made some, you know, needed changes. And that was difficult even when we were in the office...

Participant 11

Concerns regarding rules and regulations more closely align with providers seeing patients across state borders. Some providers felt such regulations hindered their ability to treat patients but also created unnecessary frustrations.

So, yeah, it’s interesting navigating all of that because, and honestly, I think it’s just so stupid having state-dependent licensing system when they all use the same criteria.

Participant 7

While providers recognized the need for laws and regulations, their current struggles often placed them in positions having to choose between seeing their (often established) patients or complying with regulations.

It reminds me of another patient, he’s in college in North Carolina and, he had really good, he had a psychiatrist through his school but then the school had canceled the contract with that [psychiatrist] and he’s like, what now am I supposed to do? And like, he wasn’t exactly, he wasn’t unstable but he wasn’t stable. Like, you know, in that gray zone and he’s like, can’t you see me?...And I would love to see you. I feel my heart goes out to you, but like, you’re in North Carolina, like, I...I, I will see you all through Christmas break, summer break. But like, I don’t know what to do. Like, if you’re having a crisis, definitely call me like, and I will, you know, I will just override it. But like, if you’re going there and, you know, like you need the help, like you gotta, I don’t know, I don’t know what to tell you. Like, I feel terrible if you.

Participant 1

Providers often reported that mobile patients such as college students are the ones most strongly affected by these types of laws and regulations.

Providers reported far fewer workarounds or solutions (27/234, 11.5% codes; 11/16, 69% providers) to mentioned barriers relating to prescribing over telemedicine. Direct responses to barriers and challenges included—as subtopics—streamlining how they receive patient health data (8/27, 30%), finding new pharmacies for patients to use (5/27, 19%), using reminders and new ways to connect with patients quickly (5/27, 19%), responding to regulatory pressures (3/27, 11%), and making individual adaptations in their practice (6/27, 22%; due to double coding of subtopics, percentages add up over 100) The most common solution providers reported was finding an easier way to receive laboratory tests, examinations, and vitals from their patients (8/27, 30% codes; 5/16, 31% providers). While providers were able to direct their patients to local laboratories or request that their patients invest in a blood pressure cuff, one provider noted their practice found certain tests to be beneficial for telemedicine visits:

Hm, we do drug screens online. So, we do saliva testing online and drug screens. And so that’s if we don’t always have to have people go pee somewhere or go to the lab, they can do those at home. So, I guess that’s something that we found just as a, not really a workaround, well, kind of a workaround, and I guess to allow them to stay at home, but just to incorporate it as something that’s a little bit more convenient.

Participant 5

Providers further noted the emphasis on streamlining patient processes by using novel ways to communicate with them regarding simple questions or requests. For example, one provider noted the following:

[Patients] can just text me a question...A few patients of mine have learned that they come and sit in the waiting room if they have a question. And so, I see them sitting in my waiting room, and sometimes I’m done. I’m like, “What's up?” And they say, “Yes, you know, hey, I need a refill.”

Participant 12

In this case, the provider sought to have secure, simple messaging with a patient. Other solutions related to streamlining processes with patients included actively talking with pharmacies in the patients’ area (5/27, 19%), moving to a new e-prescription platform (1/27, 4%), and creating a protocol for helping their out-of-state patients find health resources (1/27, 4%).

Providers mentioned two differing approaches to handling the barriers created by laws and regulations in prescribing over telemedicine. Some (2/16, 13%) providers chose to unequivocally adopt the strictest state and federal regulations within their practice, and another explained that they would continue with a process that works for their patients but was admittedly in the gray zone legally.

Content analysis of barriers affecting provider comfort in prescribing over telemedicine identified 85 code instances over 16 interviews. With 27 code instances of workarounds and solutions directly relating to these noted barriers, the qualitative analysis identifies areas in telemedicine where progress still needs to be made.

## Discussion

### Principal Findings

By leveraging the results of our more extensive mixed methods study [17], we framed our content analysis to provide needed context for understanding mental health providers’ comfort in prescribing over telemedicine. This qualitative study identified vital parameters that constitute comfort for mental health providers who prescribe via telemedicine. Our results show that providers feel most comfortable prescribing over telemedicine when they are practicing within its inherent limitations and their own professional expertise, with access to patients’ relevant health information, and within clearly defined legal and regulatory confines. In addition, we identified related barriers that affect these parameters and the potential solutions that providers have used to alleviate these challenges. Content analysis revealed providers found the e-prescription platforms and the miscommunications with pharmacies receiving prescriptions as substantial sources of frustration and concern in the prescribing process. Ultimately, providers prescribe medications over telemedicine within the bounds of their perceived comfort parameters but would like to see changes in streamlining the prescribing workflow, assurance of timely patient examinations and laboratory tests, and clarity in the laws and regulations surrounding prescribing over telemedicine.

Overall, even while providers reported feeling comfortable prescribing over telemedicine, they were aware their comfort was due to practicing within their established personal parameters. Providers noted their comfort in prescribing over telemedicine was attributable to their specialty and providing care to patients who require specific types of medications that providers may be generally hesitant to prescribe. Research using claims data between 2020 and 2023 shows that certain specialties (ie, psychiatry) were seen to initiate treatment for alcohol use disorder over telemedicine more than others (ie, primary care physicians) [22]. Within the same study, researchers observed a different rate of telemedicine initiations dependent on the types of medications being prescribed (eg, naltrexone at 14.6% vs topiramate at 11.8%). Our results support these findings as psychiatrists are better versed in treating mental health disorders and have tacit knowledge regarding certain medications and patient symptoms. Unsurprisingly, in this study, some providers emphasized that their comfort in prescribing decreased when faced with diagnoses they have little experience treating. From a 2023 review of pandemic-era research on telemedicine use in SUD treatment in the United States, authors noted providers would vary the number of days (supply) of prescribed medication based on their judgment of each patient interaction and context [23-26]. These findings are supported by our results as providers work within their personal parameters and determine how to proceed with prescribing based on their interaction with each patient.

Additionally, providers’ personal parameters likely echo current guidelines regarding telemedicine use and limitations [27,28]. Such guidelines proffered protocols and practices based on earlier telemedicine research and stricter rules and regulations [29]. Considering that guidelines emphasize compliance with state and federal laws and regulations, we may be seeing providers’ personal parameters reflecting these policies even when they feel comfortable and capable of prescribing over telemedicine [30]. Updates of such guidelines would help reframe providers’ understandings of telemedicine prescribing limitations and liabilities; however, lagging legal progress limits the ability for such revisions [31,32]. With the loosening of laws and regulations regarding telemedicine prescribing, new evidence has shown that providers can care for their patients effectively while maintaining positive patient outcomes [29].

Providers also emphasized the importance of access to patient’s health information, especially timely updates of exams and labs needed to prescribe certain medications such as stimulants. Certainly, the content analysis showed multiple workarounds focused on streamlining the process of accessing patient health information. Understandably, providers also noted that they feel more comfortable working with established patients, where patient health history and a personal repertoire with the patient can provide context to a telemedicine visit. Interview results suggest that an established patient—via telemedicine or in-person visit—is a central factor in how providers view their needs for timely patient health updates. This parameter is relevant for in-person and telemedicine visits. Nevertheless, with the inflated fear of liability when using telemedicine, providers show increased concern when operating outside their parameters for comfort in telemedicine prescribing [33]. Similar findings show providers may feel that additional assessment, such as physical examination, reinforces their decision-making and allays liability fears [34].

Our content analysis shows that the ultimate tipping point in whether providers prescribe via telemedicine is whether the clinical situation falls within the provider’s personal parameters for comfort in prescribing. The obstacles we identified in the study, such as the e-prescription platform errors, were predominantly situations that caused frustrations in the prescribing process but were insufficient reasons for providers to discontinue prescribing medications. Of course, particular barriers, such as miscommunications with pharmacies and not receiving patient examinations or laboratory tests promptly, directly impact patient safety and outcomes. Our results support prior research showing that providers and patients faced challenges with e-prescriptions where pharmacies faced a dearth of certain medications such as buprenorphine [23,35,36]. Providers emphasized the importance of patient safety with their noted solutions: the most frequently mentioned solutions revolved around streamlining the receipt of patient health information and ensuring patient access to needed medications. Given the current, typical support infrastructure in the United States, telemedicine is not as effective for treating disorders that require laboratory examinations [37]. However, telemedicine is becoming a permanent mode of health care delivery, and successful solutions for assessment, such as diagnostic testing and information access, will be a priority for providers and other stakeholders. As a result, there is a growing support infrastructure including at-home laboratory options ranging from technicians coming to your home to home tests that can be done during a telemedicine visit [38,39]. While large health institutions such as Yale School of Medicine may have such resources available for their patients [38], small clinics or solo providers face difficulties in connecting their patients to these resources, especially in relation to an individual patient’s insurance coverage [39].

Our results show that providers prescribe within the scope of their personal parameters determined by perceived limitations of telemedicine and professional expertise, provider access to relevant patient health information, and liability concerns. Once providers met their individual parameters, they expressed higher comfort in prescribing over telemedicine. With an increase in legal clarity and simplification of prescribing over telemedicine, providers may feel more capable and comfortable with the process, as guidelines would reflect the loosening of restrictions [30]. These personal parameters exist in the context of current legal and regulatory considerations; therefore, making PHE telemedicine-related policy flexibilities permanent may help expand the situations in which providers feel comfortable prescribing. Such progress may also improve patient access to mental health care services—providers may feel more comfortable expanding their patient base for telemedicine, thus alleviating access and inequity issues for mental health care treatment [40].

### Limitations

This study used interviews from providers recruited through nonprobability convenience sampling via the TelehealthEngage research panel. Our findings are not generalizable to all US mental health providers. It reflects the view of interview participants, including providers from solo, small private practices; statewide health systems and clinics; and other types of mental health care settings. Additionally, as we included all prescribing mental health providers, the prescribing challenges, workarounds, and level of comfort we captured in the study may differ for other providers based on their practice focus. Future research should include a larger sample of participants including those practicing in larger health systems and academic health sciences centers, who were underrepresented in our sample.

### Conclusions

Through content analysis of semistructured provider interviews, we determined that the participating providers feel comfortable prescribing over telemedicine when they practice within perceived limits of telemedicine and their own expertise, with sufficient access to patient health information, and when they do not have liability concerns. Providers mentioned multiple hindrances to prescribing over telemedicine. However, the ultimate reason they refer patients to other providers or convert encounters from telemedicine to in-person visits is that their comfort parameters are not met, and thus, patient safety is at risk. Future research, including inductive-deductive qualitative analysis of the interviews, will help create a more robust understanding of provider perspectives on telemedicine prescribing and inform future implementation and policy of prescribing over telemedicine.

## Appendix

Multimedia Appendix 1 Semistructured interview guide.

## Abbreviations

PHE: public health emergency
SUD: substance use disorder

## References

[1] Fiscal year 2024: budget in brief. U.S. Department of Health and Human Services.

[2] CARES Act: AMA COVID-19 pandemic telehealth fact sheet. American Medical Association.

[3] VanderWerf M, Bernard J, Barta DT, Berg J, Collins T, Dowdy M, Feiler K, Moore DL, Sifri C, Spargo G, Taylor CW, Towle CB, Wibberly KH (2022). Pandemic action plan policy and regulatory summary telehealth policy and regulatory considerations during a pandemic. Telemed e-Health.

[4] DEA hosts public listening sessions on telemedicine regulations. United States Drug Enforcement Administration.

[5] DEA extends telemedicine flexibilities for prescribing of controlled medications: second time is the charm. Foley.

[6] Huskamp HA, Riedel L, Uscher-Pines L, Busch AB, Barnett ML, Raja P, Mehrotra A (2022). Initiating opioid use disorder medication via telemedicine during COVID-19: implications for proposed reforms to the ryan haight act. J Gen Intern Med.

[7] Niyibizi A, Haveric A, Irio G (2024). Telehealth in opioid use disorder treatment: policy considerations for expanding access to care. J Osteopath Med.

[8] Hailu R, Mehrotra A, Huskamp HA, Busch AB, Barnett ML (2023). Telemedicine use and quality of opioid use disorder treatment in the US during the COVID-19 pandemic. JAMA Netw Open.

[9] Mattocks KM, Moore DT, Wischik DL, Lazar CM, Rosen MI (2022). Understanding opportunities and challenges with telemedicine-delivered buprenorphine during the COVID-19 pandemic. J Subst Abuse Treat.

[10] Lin LA, Fortney JC, Bohnert AS, Coughlin LN, Zhang L, Piette JD (2022). Comparing telemedicine to in-person buprenorphine treatment in U.S. veterans with opioid use disorder. J Subst Abuse Treat.

[11] Guillen AG, Reddy M, Saadat S, Chakravarthy B (2022). Utilization of telehealth solutions for patients with opioid use disorder using buprenorphine: a scoping review. Telemed e-Health.

[12] Hammerslag LR, Mack A, Chandler RK, Fanucchi LC, Feaster DJ, LaRochelle MR, Lofwall MR, Nau M, Villani J, Walsh SL, Westgate PM, Slavova S, Talbert JC (2023). Telemedicine buprenorphine initiation and retention in opioid use disorder treatment for medicaid enrollees. JAMA Netw Open.

[13] Uscher-Pines L, Parks AM, Sousa J, Raja P, Mehrotra A, Huskamp HA, Busch AB (2022). Appropriateness of telemedicine versus in-person care: a qualitative exploration of psychiatrists' decision making. Psychiatr Serv.

[14] Cummins MR, Ivanova J, Ong T, Soni H, Barrera JF, Wilczewski H, Welch BM, Bunnell BE (2024). Will the United States pass on telemedicine progress?. JAMIA Open.

[15] Meese KA, Hall AG, Feldman SS, Colón-López A, Rogers DA, Singh JA (2022). Physician, nurse, and advanced practice provider perspectives on the rapid transition to inpatient and outpatient telemedicine. Telemed Rep.

[16] Telemedicine can change care for the better—with the right rules. Harvard Medical School.

[17] Cummins M, Ivanova J, Soni H, Robbins Z, Bunnell BE, López E, Welch BM Telemedicine prescribing: a national, cross-sectional survey of U.S. mental health care providers. JMIR Preprints.

[18] TelehealthEngage. Doxy.me.

[19] Bernard HR, Wutich A, Ryan GW (2016). Analyzing Qualitative Data: Systematic Approaches.

[20] Campbell JL, Quincy C, Osserman J, Pedersen OK (2013). Coding in-depth semistructured interviews. Sociol Methods Res.

[21] Raskind IG, Shelton RC, Comeau DL, Cooper HLF, Griffith DM, Kegler MC (2019). A review of qualitative data analysis practices in health education and health behavior research. Health Educ Behav.

[22] Huskamp HA, Uscher-Pines L, Raja P, Normand ST, Mehrotra A, Busch AB (2024). Telemedicine for initiation of alcohol use disorder medications. JAMA Netw Open.

[23] Lin C, Pham H, Zhu Y, Clingan SE, Lin L, Murphy SM, Campbell CI, Sorrell TR, Liu Y, Mooney LJ, Hser Y (2023). Telemedicine along the cascade of care for substance use disorders during the COVID-19 pandemic in the United States. Drug Alcohol Depend.

[24] Uscher-Pines L, Sousa J, Raja P, Mehrotra A, Barnett M, Huskamp HA (2020). Treatment of opioid use disorder during COVID-19: experiences of clinicians transitioning to telemedicine. J Subst Abuse Treat.

[25] Nordeck CD, Buresh M, Krawczyk N, Fingerhood M, Agus D (2021). Adapting a low-threshold buprenorphine program for vulnerable populations during the COVID-19 pandemic. J Addict Med.

[26] Duncan A, Sanders N, Schiff M, Winkelman TN (2021). Adaptations to jail-based buprenorphine treatment during the COVID-19 pandemic. J Subst Abuse Treat.

[27] Smith K, Ostinelli E, Macdonald O, Cipriani A (2020). COVID-19 and telepsychiatry: development of evidence-based guidance for clinicians. JMIR Ment Health.

[28] Gough F, Budhrani S, Cohn E, Dappen A, Leenknecht C, Lewis B, Mulligan DA, Randall D, Rheuban K, Roberts L, Shanahan TJ, Webster K, Krupinski EA, Bashshur R, Bernard J (2015). ATA practice guidelines for live, on-demand primary and urgent care. Telemed e-Health.

[29] Ivanova J, Ong T, Wilczewski H, Cummins M, Soni H, Barrera J, Welch B, Bunnell B Telemental health care guidelines from the first 2 years of the COVID-19 pandemic: a scoping review using content and thematic analyses. JMIR Preprints.

[30] Krawczyk N, Rivera BD, King C, Dooling BCE (2023). Pandemic telehealth flexibilities for buprenorphine treatment: a synthesis of evidence and policy implications for expanding opioid use disorder care in the United States. Health Aff Sch.

[31] Double secret protection: bridging federal and state law to protect privacy rights for telemental and mobile health users. Duke Law Journal.

[32] Tong KW (2019). Telehealth as a double-edged sword: lessons from court cases to gain understanding of medico-legal risks. Med Law.

[33] Nguyen M, Waller M, Pandya A, Portnoy J (2020). A review of patient and provider satisfaction with telemedicine. Curr Allergy Asthma Rep.

[34] Riley PE, Fischer JL, Nagy RE, Watson NL, McCoul ED, Tolisano AM, Riley CA (2021). Patient and provider satisfaction with telemedicine in otolaryngology. OTO Open.

[35] Castillo M, Conte B, Hinkes S, Mathew M, Na CJ, Norindr A, Serota DP, Forrest DW, Deshpande AR, Bartholomew TS, Tookes HE (2020). Implementation of a medical student-run telemedicine program for medications for opioid use disorder during the COVID-19 pandemic. Harm Reduct J.

[36] Tofighi B, McNeely J, Walzer D, Fansiwala K, Demner A, Chaudhury CS, Subudhi I, Schatz D, Reed T, Krawczyk N (2022). A telemedicine buprenorphine clinic to serve New York City: initial evaluation of the NYC public hospital system's initiative to expand treatment access during the COVID-19 pandemic. J Addict Med.

[37] Haleem A, Javaid M, Singh RP, Suman R (2021). Telemedicine for healthcare: capabilities, features, barriers, and applications. Sens Int.

[38] Corcoran TP Mobile lab helps Yale pathology establish relationships, build trust with community. Yale School of Medicine.

[39] Drake K At-home blood tests are becoming a trend. What it means for healthcare. Healthline.

[40] Garett R, Young SD (2023). Potential effects of digital inequality on treatment seeking for opioid use disorder. Int J Mental Health Addict.

