# Supplementary material for: Mental Health Providers’ Challenges and Solutions in Prescribing Over Telemedicine: Content Analysis of Semistructured Interviews
Source: JMIR Hum Factors. 2025 Mar 20;12:e65419. doi: 10.2196/65419 (PMC11969126; doi:10.2196/65419)
Supplement: Multimedia Appendix 1 [file humanfactors_v12i1e65419_app1.docx]

[Recording begins, verbal informed consent given by participant]

Firstly, I want to thank you for your willingness to participate in our interviews. Without your time and participation, we cannot understand providers’ needs and experiences. The work we do is with the intent to develop evidence-based practices or interventions to help providers and patients. As you know from taking the survey, today we’re interested in understanding your experiences with prescribing over telemedicine.

1. [getting an idea of the provider] I would like to get to know more about you and your practice. Could you tell me a bit about your specialty, how many patients you see a day/week on average, and anything that may be unique to your practice?
   1. Prompt: how have you included telemedicine in your practice?
   2. Prompt: How has telemedicine changed the way you practice?
   3. Prompt: Do you feel your patients have different expectations from telemedicine than in-person visits?
2. [barriers] Thinking over the past 3 months with your patients, could you tell me about difficulties you have had in prescribing medication over telemedicine?
   1. [considering regulatory changes] Would you say that these difficulties have changed since the end of the public health emergency? (Differences from 6 months ago, 1 year ago, pre-COVID)
   2. [considering specific medical scenarios, including drug type] Do you feel there are certain cases that make prescription more difficult for you?
      1. Please elaborate
         1. Prompt: A specific type of illness? Medication? a type of patient?
         2. Prompt: Based on what you discussed just now, what has been your experience with medication-assisted treatment over telemedicine for SUD treatment?
         3. [pinpoint biggest hurdle source] Do you feel that the case you explained is more difficult over telemedicine because of the standard of care? regulations in place on a federal or state level? other reasons?
      2. Do you feel that your colleagues run into similar issues?
   3. [the impact of barriers to the ability to extend care] Do you feel these difficulties have made you less likely to prescribe over telemedicine?
   4. [perspective on patients] What do your patients think about some of these
3. Considering what you just talked about, could you give me an example of how you have had to adapt a typical in-person workflow to fit your telemedicine workflow instead?
   1. What do you think made this workflow successful?
   2. Prompt: Did you have to go through multiple iterations to get to this adaption you described?
4. [picking up on any workaround, workflows, services, resources] Thinking back over the past three months, what do you think has been most helpful in your ability to effectively prescribe medications over telemedicine?
   1. Prompt: Can you think of anything particular to physical assessments that has been especially helpful? What about for patient medical history? Laboratory tests? Overall coordination of care with other providers?
   2. Are there any (other) resources or services you feel have helped you in being able to prescribe over telemedicine?
5. [compliance knowledge] Do you feel that you have a good understanding of the laws and regulations surrounding prescribing medications over telemedicine, specifically in your field?
   1. Prompt: On a scale of 1 to 5, how would you rate your knowledge on this topic with one having no knowledge and five being the most knowledgeable?
   2. With the end of the PHE and upcoming, expected changes to regulations and rules in prescribing over telemedicine, could you tell me a bit about your understanding of general prescribing medication over telemedicine? medication-assisted treatment for SUD treatment? specific classifications of drugs and a provider’s ability to prescribe them?
   3. [considering modes of dispensing information effectively] What has been the most useful way to gather information regarding laws and regulations for you?
   4. [considering what makes a source credible and trustworthy] Why do you feel you can trust [the source(s)]?
6. Finally, thinking about the conversation we just had, if you could give a new provider advice on how to effectively, and successfully prescribe over telemedicine, what would you tell them?
   1. Prompt: would you recommend trying to prescribe medication over telemedicine? Is there something you would warn them about?
7. Before we conclude, is there anything you might want to share with us about your experience that I haven’t asked?

I want to thank you, once again, for your time and willingness to participate in our research study.

[stop recording, collect email for egift card]
